# Editorial: Demystifying academic writing in higher education: a process view on academic textual production

**DOI:** 10.3389/fpsyg.2025.1702487

**Published:** 2025-09-30

**Authors:** Xinghua Liu, Rui A. Alves, A. Angelique Aitken, Josef Schmied

**Affiliations:** ^1^Shanghai Jiao Tong University, Shanghai, China; ^2^University of Porto, Porto, Portugal; ^3^The Pennsylvania State University (PSU), University Park, PA, United States; ^4^Chemnitz University of Technology, Chemnitz, Germany

**Keywords:** academic writing, higher education, process view, teaching and learning of writing, AI

Writing is not an innate human ability but a skill acquired through training and sustained practice. Nonetheless, it plays a central role in how students learn and how their learning is assessed. Academic writing, where students communicate scholarly ideas, presents unique challenges. At the higher education level, strong academic writing requires clarity of reasoning, mastery of disciplinary knowledge, and linguistic proficiency.

Decades of research have deepened our understanding of the textual features of students' academic writing and the cognitive processes involved. However, a comprehensive process-oriented perspective on academic writing remains underdeveloped. Therefore, the purpose of this Topical Research is to explore how higher education students generate ideas, draft their texts, utilize technology, sustain academic integrity, and finalize their written work.

This Research Topic features four contributions examining the linguistic features of academic writing. A key characteristic of successful academic writing is the construction of authorial identity. Tian and Liu's systematic review reveals that over the past three decades, the most prominent Research Topics have centered on plagiarism/academic integrity and sociocultural perspectives on identity construction. Their findings hold particular relevance in the current AI-driven era, where the use of AI writing tools has become ubiquitous.

Through corpus-based linguistic analysis, Dudău et al. identified distinct emotional patterns in Romanian vs. English academic writing. They found that Romanian texts consistently exhibited greater formality and indirectness, which they believe have been shaped by language, cultural norms, and academic conventions.

Gong et al. investigated citation practices among novice and expert authors in the field of Chinese Applied Linguistics. Their study found striking similarities between the two groups, with minimal cross-linguistic differences in citation practices between English and Chinese academic writing.

By using a Bayesian network approach, Singh et al. modeled the cognitive processes of argumentation. Their research highlights students' primary challenges during argumentative writing, namely the framing of counterarguments and the development of in-depth and critical analyses of problems.

Feedback is an integral component of teaching and learning writing, and this Research Topic includes two papers addressing this topic. Liu and Xin explored the emotional responses of Chinese as a Foreign Language (CFL) learners when receiving oral and written feedback from their teachers, and examined their emotion regulation strategies as well. Their findings reveal that teacher feedback elicits three types of emotions: academic achievement emotions, cognitive emotions, and social emotions. The study also highlights that students employ three primary strategies to manage negative emotions: emotion-oriented, appraisal-oriented, and situation-oriented approaches. Wei and Liu conducted a systematic review of peer feedback research in academic writing from 2014 to 2024. They identified five key benefits of peer feedback activities: affective, cognitive, behavioral, social, and meta-cognitive benefits. Additionally, they pinpointed three major challenges associated with peer feedback: difficulties arising from feedback providers, receivers, and contextual factors.

Writing is a cognitively demanding task and presents additional challenges for students learning to write in a foreign language. Therefore, cultivating and sustaining students' motivation is crucial for the success of academic writing instruction. This Research Topic includes two studies on writing motivation. Abdel Latif et al. surveyed experienced English writing teachers from five Saudi universities, identifying eight effective motivational strategies, such as optimizing teacher feedback, negotiating writing topic choices. Their findings also suggest that smaller class sizes facilitate the implementation of these strategies. For doctoral students, mastering academic writing is particularly critical, as it serves as the primary gateway to the academic community. Becker et al. employed a comparative case study approach to examine online mentoring dynamics. They identified five key factors that can help build trust and collaboration between supervisors and research students.

The pervasive influence of AI has made it imperative to integrate AI technology into academic writing instruction. This Research Topic includes two relevant contributions on this topic. Zhang's study contributes to the growing evidence supporting AI-assisted writing instruction. The research demonstrates that when AI tools are used in a guided, structured manner, university students report improved writing quality, enhanced perceived mental wellbeing, and greater academic engagement. Wang's questionnaire survey of Chinese EFL students reveals key insights into their use of large language models (LLMs) for business English writing. The findings indicate that performance expectancy and social influence strongly predict students' intention to use LLMs. What is particularly interesting is that motivation not only influences students' perception of the usefulness of LLMs, but also determines students' actual use of them in their writing processes.

The papers in this Research Topic explore diverse aspects of academic writing in higher education, demonstrating both the richness and complexity of this field. While significant progress has been made, we identify three critical areas requiring further investigation. First, the planning, composing, and revision stages of academic writing remain largely unexplored. A deeper understanding of students' challenges and effective instructional strategies is urgently needed. Second, as AI is transforming education at all levels, it is imperative to study how we can maximize AI's benefits for teaching and learning writing while mitigating its potential risks and ethical concerns. Third, we believe good academic writing instruction needs to help develop autonomous writers. While more research is warranted for developing students' self-regulation skills and equipping them with the independence needed for lifelong academic success, the articles collected here are already insightful pointers in that direction.

